# Conservative Management of Suspected Emphysematous Gastritis

**DOI:** 10.7759/cureus.31995

**Published:** 2022-11-28

**Authors:** Thomas G Mackay, Du H Phan, Pranay S Mantha, Hany S Ibrahim, Matthew J Burstow

**Affiliations:** 1 Department of Surgery, Logan Hospital, Logan, AUS; 2 School of Medicine and Dentistry, Griffith University, Gold Coast, AUS

**Keywords:** conservative vs surgical management, gastritis, gastric pneumatosis, emphysematous gastritis, gastric emphysema

## Abstract

Emphysematous gastritis is a rare surgical condition. Although there is a lack of a common definition, the key features of its presentation include gastric emphysema on imaging and the presence of gas-forming organisms in the gastric mucosa. In this study, we report the case of an 80-year-old Caucasian male who presented with abdominal pain; a computed tomography scan demonstrated gastric emphysema (intra-mural air within the stomach). After upper gastrointestinal endoscopy excluded gross perforation, ulcer, and malignancy, the patient recovered to baseline with conservative management consisting of gastric rest (nil by mouth and nasogastric tube decompression), intravenous antibiotics, and intravenous proton pump inhibitor. Given the wide pathogenic mechanisms for gastric emphysema, we recommend a conservative but cautious approach if the patient does not demonstrate clinical features of hemodynamic instability, sepsis, and peritonitis.

## Introduction

Emphysematous gastritis is an infrequently observed phenomenon, with fewer than 200 prior reports in the literature. There is currently no consensus on the definition of emphysematous gastritis; however, its predominant characteristics include the features of gastric emphysema on imaging as well as microscopy and/or culture demonstrating gas-forming organisms in the gastric mucosa [1]. Gastric emphysema or pneumatosis (not to be confused with emphysematous gastritis) is a radiological finding with various mechanisms to explain its presence [2], and gastric emphysema alone is considered a relatively benign finding [3]. Ischaemia secondary to hypoperfusion is reported as the most common cause, followed by disruption of gastric mucosa, intramural infection by gas-forming organisms, and dissection of mediastinal air [4]. Given that there is wide potential pathogenesis for this finding, a cautious initial management approach is warranted.

## Case presentation

**Figure 1:**
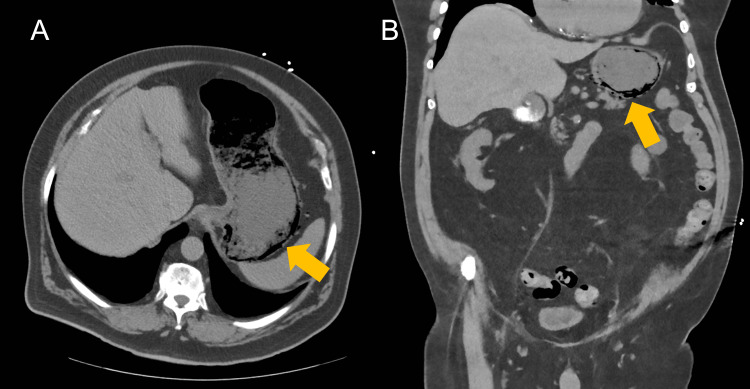
(A) Axial and (B) coronal CT abdomen images demonstrating gastric emphysema of the posterior wall and fundus of the stomach. The arrows indicate the areas demonstrating gastric emphysema. CT: computed tomography

An 80-year-old Caucasian male with a medical history of ischaemic heart disease, well-controlled insulin-dependent diabetes mellitus, and hypertension (Stage I) presented to the emergency department of our metropolitan hospital with pre-syncopal symptoms and diarrhea for two weeks. The patient had an upper gastrointestinal (UGI) endoscopy 16 months prior to this presentation for chronic cough, demonstrating mild gastritis but no helicobacter pylori infection on biopsy. On arrival, he was found to have lower abdominal pain, and observations showed he was hypotensive (116/56 mmHg, normal), bradycardic (55 beats per minute), and afebrile (temperature 36.4°C) and had a respiratory rate of 19 breaths per minute and normal pulse oximetry (saturation of 99% on room air). The patient had a soft but distended abdomen with mild lower abdominal tenderness and no features of peritonitis. Laboratory investigations on arrival showed hemoglobin of 144g/L, elevated white cell count (11.4 × 109/L) with eosinophilia, and normal electrolytes. Since his venous blood gas demonstrated a normal anion gap metabolic acidosis with normal serum lactate, combined with a negative urinalysis and persistent abdominal pain, a computed tomography (CT) scan of the abdomen was deemed necessary. Owing to the global contrast shortages, a non-contrast scan was performed. The CT of the abdomen revealed a distended stomach with intra-mural gas throughout the posterior wall and fundus, along with mild peri-gastric fat stranding (Figure 1). There was no inflammatory process nor pneumatosis demonstrated in any small or large bowel segments and no evidence of pneumoperitoneum.

**Figure 2:**
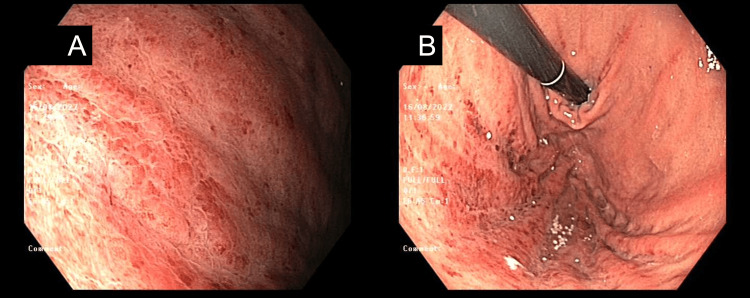
UGI endoscopy image demonstrating severe gastritis of the (A) posterior aspect of the greater curvature and (B) the fundus of the stomach. UGI: Upper gastrointestinal

The patient was made nil by mouth, underwent decompression through the insertion of a nasogastric tube, was resuscitated with intravenous fluids, and was administered intravenous piperacillin/tazobactam and intravenous pantoprazole infusion until an urgent UGI endoscopy was performed. The UGI endoscopy demonstrated severe gastritis of the posterior aspect of the greater curvature (Figure 2A) and at the fundus of the stomach (Figure 2B). There was no gross perforation, ulcer, or features of malignancy. Biopsy demonstrated active chronic gastritis and eosinophilia without helicobacter pylori or parasitic infection. The patient experienced a gradual diet upgrade and was discharged home on an oral proton pump inhibitor on the second post-endoscopy day. This patient was managed in August 2022. Till the time of submission, no complication or representation had been observed.

## Discussion

Various case reports have associated high mortality with infective causes of gastric emphysema, especially in the case of concurrent advanced age, abdominal surgical history, malnutrition, immunosuppression, and features of systemic inflammatory response syndrome [4]. Serum lactate >2 mmol/L and creatinine > 132 umol/L are also predictors of mortality [5]. While UGI endoscopy is useful in confirming the diagnosis of gastritis and other luminal pathologies, its use as a diagnostic procedure may potentiate complications, including gastric or intestinal perforation, due to inflammation and weakness of the luminal wall [6].

In this case, the patient’s dehydrated state from a prolonged diarrhoeal illness as well as the presence of vascular disease risk factors, lack of evidence of local or systemic infection with gas-forming organisms, and prompt response to conservative management indicate transient hypoperfusion and mucosal ischemia are the probable causes. Although the biopsy results related to the involved gastric mucosa demonstrated gastritis without infective organisms, it is worthwhile to note that at least 48 hours of broad-spectrum antibiotic had been administered prior to the UGI endoscopy, and thus, a diagnosis of “emphysematous gastritis” as per the definition above cannot be excluded.

The patient’s rapid response to intravenous fluid resuscitation and overall clinical improvement contributed to the decision for early UGI endoscopy. Surgery was avoided in this case, but previously, it was thought that gastric emphysema necessitates operative management, especially when there are signs of sepsis, peritonitis, and/or the presence of portal venous gas [7]. Recently, however, it appears that reported cases have demonstrated favorable outcomes with conservative management alone, which is consistent with the present case [8,9]. In the absence of sepsis, it is also suggested that intravenous antibiotics are not required, and other adjuvants such as parenteral nutrition can aid in management, thus avoiding the morbidity of operative management [8,10].

## Conclusions

Emphysematous gastritis, although uncommon, has historically been associated with high morbidity and mortality; thus, it is an important diagnostic consideration for patients presenting with gastric emphysema on a CT scan. We recommend that, in the absence of marked hemodynamic instability, sepsis, and features of peritonitis, a conservative management approach should be taken. Despite the lack of consensus on the definition of emphysematous gastritis, surgeons should be cognizant of gastric emphysema as well as the spectrum of etiology and clinical presentations in this diagnosis and tailor their approach accordingly.
